# Erosions in the foot at baseline are predictive of orthopaedic shoe use after 10 years of treat to target therapy in patients with recent onset rheumatoid arthritis

**DOI:** 10.1007/s10067-015-3145-1

**Published:** 2015-12-22

**Authors:** Sytske Anne Bergstra, Iris M. Markusse, Gülşah Akdemir, H. Karel Ronday, K. Huub Han, Willem F. Lems, Pit J. S. M. Kerstens, Rosaline van den Berg, Robert B. M. Landewé, Cornelia F. Allaart

**Affiliations:** 1 Department of Rheumatology, Leiden University Medical Center, P.O. Box 9600, 2300 RC Leiden, The Netherlands; 2 Department of Rheumatology, Haga-Leyenburg Teaching Hospital, The Hague, The Netherlands; 3 Department of Rheumatology, Maasstad Hospital, Rotterdam, The Netherlands; 4 Department of Rheumatology, VU Medical Center, Amsterdam, The Netherlands; 5 Department of Rheumatology, Reade, Amsterdam, The Netherlands; 6 Amsterdam Rheumatology & Immunology Center, Academic Medical Center Amsterdam, Amsterdam, The Netherlands; 7 Amsterdam Rheumatology & Immunology Center, Atrium Medical Center Heerlen, Heerlen, The Netherlands

**Keywords:** Erosions, Orthopaedic shoes, Rheumatoid arthritis, Risk factors

## Abstract

The objective of this study is to investigate if foot joint damage due to rheumatoid arthritis (RA) can predict whether patients will start wearing orthopaedic shoes (OS) within 10 years after treatment start. Data from recent onset RA patients with 10 years follow-up from the BeSt (Dutch acronym for treatment strategies) study were used. Treatment was tightly controlled, targeted at disease activity score (DAS) ≤2.4, according to 1 of 4 pre-specified treatment strategies. After 10 years of follow-up, orthopaedic shoe use was recorded in 285/508 patients (responders to questionnaires at 10 years). Between-group differences for orthopaedic shoe users and non-users were calculated at baseline, after 10 years, and change scores over the 10-year period were calculated. Predictors for orthopaedic shoe use were identified by univariable and multivariable logistic regression analyses. Orthopaedic shoe use was reported by 57/285 patients after 10 years. Orthopaedic shoe users had more joint damage, joint swelling and pain in the feet already at baseline and after 10 years. At both time points, DAS of orthopaedic shoe users and non-users was similar. Multivariable logistic regression showed that dichotomized foot erosions score (cut-off ≥1 erosion) (OR 2.42), anti-citrullinated protein antibodies (ACPA) (OR 4.64) and DAS (OR 1.77) were independent predictors of orthopaedic shoe use. Despite intensive targeted treatment, 57/285 recent onset RA patients started using orthopaedic shoes over 10 year of follow-up. Presence of foot erosions at treatment start predicts orthopaedic shoe use after 10 years. The risk of orthopedic shoe use increased for ACPA-positive patients and for those with higher baseline disease activity.

**Electronic supplementary material:**

The online version of this article (doi:10.1007/s10067-015-3145-1) contains supplementary material, which is available to authorized users.

## Introduction

Despite major improvements in the treatment of rheumatoid arthritis (RA) patients, joint damage is still a common disease manifestation. The presence of erosions in an early disease stage is indicative of a potentially severe disease course with further joint damage progression [1, 2]. Several authors have reported that in recent onset RA patients, erosions and joint space narrowing (JSN) occur more frequently in the feet than in the hands, particularly in the metatarsophalangeal joints [2–5].

Up to 80 % of RA patients reported disease-related feet problems, often already in an early disease stage (53 % of patients) [6]. Reported symptoms include pain, stiffness, swelling and ultimately deformities of the foot joints with or without overlying ulcers [7, 8]. Symptoms of the feet may disturb the gait pattern, thereby negatively affecting mobility and health-related quality of life [8, 9].

It has been shown that wearing custom-made orthopaedic shoes (OS) is associated with pain reduction and improved mobility and activity participation [10, 11]. Previously, 25 to 30 % of RA patients with advanced RA reported OS use, which may underrepresent OS need, as many patients dislike OS [12, 13]. Ideally, effective treatment of RA should prevent a need for OS. We hypothesized that in an intensely monitored, treated to target cohort of RA patients, such as participants of the BeSt study, the need for OS would be decreased compared to historic cohorts and investigated whether foot joint damage due to RA can predict OS use in patients who completed 10 years follow-up.

## Materials and methods

### Patients

Data from the BeSt (Dutch acronym for treatment strategies) study were used. The BeSt study has been described extensively before [14]. In short, it is a multicenter randomized trial (Dutch trial registry, NTR262 and NTR265) in which 508 recent onset RA patients (1987 American College of Rheumatology criteria [15]) were treated during a 10-year follow-up period. Patients were randomized into one of four treatment strategies: sequential monotherapy, step-up combination therapy, initial combination therapy with prednisone or initial combination therapy with infliximab. Treatment was adjusted if the three-monthly measured disease activity score in 44/53 joints (DAS) was >2.4. The Medical Ethical Committees of all participating centres approved both the study protocol and a separate protocol to approach all patients to fill out an additional questionnaire at 10 years. All patients gave written informed consent for the original study, and patients who filled out the questionnaire had signed a separate written informed consent.

At baseline and each following year, radiographs of the hands and feet were made. Two trained readers, unaware of the treatment strategy and patient data, have independently read the radiographs in random time order using the Sharp/van der Heijde score (SHS) [16]. The final score was the two readers’ mean score. At baseline, extensive disease measures [e.g., DAS, Health Assessment Questionnaire (HAQ) [17], erythrocyte sedimentation rate (ESR), Ritchie Articular Index (RAI), swollen joint count (SJC) in 66 joints] and patient and disease characteristics [e.g., IgM rheumatoid factor (RF), anti-citrullinated protein antibodies (ACPA), smoking status (yes/no) and body mass index (BMI)] were collected. Every 3 months, disease activity measures and disease characteristics were obtained.

After 10 years follow-up, all original participants were asked by a questionnaire if they were currently using OS (either custom-made or off the shelf). Current use of OS, but not the time of first use, was recorded. Since all patients included in the BeSt study had recent onset RA (median 2 weeks from diagnosis to inclusion), it was assumed that none used OS at baseline.

### Statistical methods

Patients using OS were compared to non-users regarding patient and disease characteristics at baseline and at year 10. Change scores over the follow-up period were calculated and compared between OS users and non-users. Between-group differences for categorical variables were analysed using *χ*^2^ tests. Normally distributed variables were analysed using *t* tests and non-normally distributed variables were analysed using Mann–Whitney *U* tests. The four treatment strategies as well as mono- vs combination therapy groups were compared on the number of OS users (*χ*^2^ tests). Logistic regression analyses were performed with OS use as binary outcome variable.

To avoid bias due to missing data in the logistic regression analyses, multiple imputation was performed for the predictor variables containing missing data (the continuous variables foot JSN and foot erosions and the dichotomous variable ACPA) using a multivariate normal model [18] with 50 imputation cycles.

In the logistic regression, a maximum of 1 predictor per 10 patients (outcome OS use) was modelled. First, univariable logistic regression was performed including baseline foot erosions and foot JSN as potential predictors. Since in the BeSt population, only few patients show severe radiographic damage, the distribution of the foot damage variables is highly skewed with many ‘zeros’ and relatively few high scores. Therefore, foot erosions and foot JSN were added as dichotomized predictors (cut-off value ≥1), in order to better approximate this ‘zero-inflated model’.

Predictors with a *p* value <0.1 were added for further testing in a multivariable model. Next, age, BMI, smoking status, baseline DAS and a combination of ACPA and RF (four categories) were simultaneously added in a multivariable model, in combination with the contributory variables from the univariable regression. The selection of these variables was based on previous research [17–19]. The HAQ was not added as a predictor, due to its high correlation with the DAS.

Only predictors with statistically significant contribution were kept in the final model. Possible interaction between treatment arm and each predictor was tested in a logistic regression analysis with OS use as outcome variable. In case of statistically significant interaction (*p* < 0.05), OS use should be analyzed per treatment arm. In case of no interaction, patients in all treatment arms were analyzed together. All analyses were performed using State SE version 14 (StataCorp LP).

## Results

After 10 years, 307/508 patients (60 %) were still participating in the BeSt study. In total, 279/307 filled out the questionnaire about OS use at year 10. Patients had dropped out due to several reasons: 76 patients refused to continue participation, 9 had a revised diagnosis (no RA), 35 had a comorbidity contraindicating the pre-specified treatment, 42 had another reason (not further specified) and 39 died. Six patients who had dropped out agreed to fill out the questionnaire. In total, information about OS use after 10 years was available in 285/508 patients (56 %).

Responders were younger than non-responders (mean age 51.3 (SD 12.0) vs. 58.4 (SD 14.8) years, *p* < 0.001) and had a lower HAQ (mean 1.3 (SD 0.6) vs. 1.5 (SD 0.7), *p* = 0.020) but did not differ statistically significantly on gender, smoking status, ACPA positivity, baseline DAS, total SHS, total JSN and total erosion scores (Supplementary Table 1).

Twenty percent of the responders (57/285) reported to use OS. Baseline characteristics of OS users and non-users are presented in Table 1. OS users were statistically significantly more often ACPA positive and more frequently had erosions at baseline. They also had a statistically significantly higher total RAI, a higher RAI of the foot and ankle joints, a higher SJC of the foot and ankle joints and a higher total joint erosions score, foot erosions score and hand JSN score. The distribution of OS users and non-users over the allocated treatment groups did not significantly differ (Pearson *χ*^2^(3) = 6.6, *p* = 0.085). Also, the proportion of patients in the arms grouped together as mono- or combination therapy did not significantly differ (Pearson *χ*^2^(1) = 2.03, *p* = 0.154).

**Table 1:** Baseline characteristics for orthopaedic shoe users (*n* = 57) and non-users (*n* = 228)

	Orthopaedic shoe users		Non-orthopaedic shoe users		
	*N*		*N*		*p* value
Age years	57	50.3 (11.0)	228	51.6 (14.8)	0.496
Female (%)	57	68.4	228	67.1	0.877
BMI (kg/m^2^)	57	26.4 (3.3)	228	26.0 (3.8)	0.503
Smoking (% yes)	57	38.6	228	29.8	0.567
Alcohol consumption (% yes)	57	56.1	227	51.3	0.181
Rheumatoid factor positive (%)	57	77.2	228	65.8	0.099
ACPA positive (%)	54	83.3	223	57.0	<0.001
CRP [median (range)]	57	20 (0; 172)	223	21 (0; 237)	0.895
DAS	57	4.6 (0.9)	228	4.4 (0.9)	0.115
Ritchie articular index	57	15.6 (7.3)	227	13.6 (6.6)	0.049
Total SJC	57	15.3 (7.2)	227	14.5 (6.3)	0.385
TJC foot and ankle joints [median (range)]	56	12 (0; 26)	226	10 (0; 30)	0.053
SJC foot and ankle joints [median (range)]	56	6 (0; 16)	222	4 (0; 21)	0.033
ESR [median (range)]	57	33 (3; 97)	228	36.5 (2; 143)	0.843
HAQ	57	1.5 (0.7)	228	1.3 (0.6)	0.146
VAS pain (mm)	57	56.2 (18.4)	227	53.4 (22.2)	0.381
VAS general health (mm)	57	54.3 (19.1)	228	51.3 (20.5)	0.308
VAS disease activity (mm)	57	61.5 (19.8)	227	59.7 (23.2)	0.581
VAS morning stiffness (mm)	57	58.1 (21.5)	227	60.7 (24.6)	0.462
Patients with erosions (%)	55	40.4	222	24.1	0.017
Total SHS [median (range)]	55	3 (0; 21.5)	213	1.5 (0; 35.5)	0.080
Bone erosions [median (range)]	55	0 (0; 14)	213	0 (0; 20.5)	0.030
Bone erosions foot [median (range)]	56	0 (0; 14)	215	0 (0; 20.5)	0.038
Bone erosions hands [median (range)]	56	1 (0; 3.5)	218	0 (0; 9.5)	0.060
JSN [median (range)]	55	2 (0; 16.5)	214	1.5 (0; 19.5)	0.193
JSN foot [median (range)]	56	0 (0; 8.5)	217	0 (0; 15)	0.170
JSN hands [median (range)]	56	1 (0; 16.5)	217	1 (0; 34)	0.025

Mean (SD) reported if not stated otherwise

*ACPA* anti-citrullinated protein antibodies, *BMI* body mass index, *DAS* disease activity score, *TJC* tender joint count, *SJC* swollen joint count, *ESR* erythrocyte sedimentation rate, *HAQ* health assessment questionnaire, vas visual analogue scale, *CRP* C-reactive protein, *SHS* sharp/van der Heijde score, *JSN* joint space narrowing

In Table 2, patient and disease characteristics at year 10 as well as change scores over the 10-year follow-up period are shown. OS users more often had a higher HAQ, a higher SJC of the foot and ankle, as well as a higher total SHS and all of its sub-scores, except for hand erosions. The OS users also showed greater increase in total SHS and all of its sub-scores, except for hand erosions.

**Table 2:** Ten years data and change in patient and disease characteristics between baseline and 10 years for orthopaedic shoe users (*n* = 57) and non-users (*n* = 228)

	Orthopaedic shoe users		Non-orthopaedic shoe users		*p* value
	*N*		*N*		
BMI (kg/m^2^)	56	26.7 (4.3)	208	26.6 (4.2)	0.917
Δ BMI (kg/m^2^)	56	0.2 (0.3)	208	0.6 (2.5)	0.304
Smoking (% yes)	56	33.9	208	22.1	0.069
Δ Smoking	56	−4.8	208	−7.7	0.936
Alcohol consumption (% yes)	55	60.0	207	63.0	0.685
Δ Alcohol consumption	55	3.9	207	11.6	0.551
CRP [median (range)]	55	3 (1; 52)	210	3 (0; 147)	0.752
Δ CRP	55	−15 (−172; 36)	205	−16 (−231; 136)	0.749
DAS	55	1.7 (0.8)	216	1.6 (0.7)	0.440
Δ DAS	55	−2.9 (1.1)	216	−2.7 (1.0)	0.347
Ritchie articular index	56	2.5 (3.6)	217	1.9 (2.5)	0.064
Δ Ritchie articular index	56	−13.2 (7.3)	216	−11.5 (6.4)	0.097
Total SJC	55	1.2 (2.2)	218	1.4 (2.3)	0.031
Δ Total SJC	55	−14.3 (7.4)	217	−13.0 (6.9)	0.222
TJC foot/ankle joints [median (range)]	56	12 (0; 26)	226	10 (0; 30)	0.053
Δ TJC foot/ankle joints	53	−8 (−24; 46)	210	−7 (−30; 20)	0.364
SJC foot/ankle joints [median (range)]	56	6 (0; 16)	222	4 (0; 21)	0.033
Δ SJC foot/ankle joints	55	−5 (−16; 0)	212	−3.5 (−21; 7)	0.076
ESR [median (range)]	54	18.4 (17.4)	217	20.1 (18.0)	0.337
Δ ESR	54	−20.5 (−75; 31)	217	−14 (−105; −52)	0.586
HAQ	56	0.8 (0.7)	217	0.5 (0.5)	0.005
Δ HAQ	56	−0.7 (0.7)	217	−0.8 (0.7)	0.496
VAS pain (mm)	56	23.7 (23.6)	216	19.6 (20.9)	0.205
Δ VAS pain	56	−32.5 (27.8)	215	−33.4 (25.1)	0.809
VAS general health (mm)	56	24.4 (18.8)	217	21.6 (20.9)	0.368
Δ VAS general health	56	−30.59 (26.4)	217	−29.4 (25.5)	0.753
VAS disease activity (mm)	56	24.3 (22.3)	217	20.3 (21.1)	0.223
Δ VAS disease activity	56	−37.68 (28.0)	216	−39.0 (27.3)	0.741
VAS morning stiffness (mm)	56	23.2 (22.7)	216	20.5 (20.8)	0.382
Δ VAS morning stiffness	56	−34.3 (27.3)	215	−40.4 (26.7)	0.135
Patients with erosions (%)	55	75.4	225	58.8	0.020
Δ Patients with erosions	55	35.1	225	34.7	0.593
Total SHS [median (range)]	49	13.5 (0; 146.5)	206	6 (0; 224.5)	0.003
Δ Total SHS	47	3.5 (0.0; 145.5)	193	2.00 (−1.5; 215.5)	0.007
Bone erosions	49	4 (0.0; 54.5)	206	1 (0; 98.5)	0.008
Δ Bone erosions	47	1.5 (0.0; 50)	192	0.05 (−0.05; 97)	0.009
Bone erosions foot [median (range)]	51	1.5 (0.0; 42.5)	209	0 (0; 32.5)	0.002
Δ Bone erosions foot	50	1.0 (0.0; 28.5)	197	0.0 (−0.5; 31.0)	0.002
Bone erosions hands [median (range)]	51	0.5 (0; 37.5)	208	0 (0; 66)	0.061
Δ Bone erosions hands	50	0.25 (−0.05; 37.5)	199	0.0 (−0.5; 66)	0.196
JSN [median (range)]	50	8.25 (0.0; 96.5)	207	4 (0; 126)	0.004
Δ JSN	48	2.75 (−1.5; 95.5)	195	1.0 (−1.5; 118.5)	0.007
JSN foot [median (range)]	52	2 (0.0; 36)	210	0 (0; 40)	<0.001
Δ JSN foot	51	1.0 (0.0; 36.0)	200	0.0 (0.0; 35.0)	<0.001
JSN hands [median (range)]	51	4.5 (0.0; 60.5)	208	3 (0; 86)	0.025
Δ JSN hands	50	1.25 (−1.5; 59.5)	199	0.0 (−1.5; 83.5)	0.037

Mean (SD) reported if not stated otherwise

*RF* rheumatoid factor, *ACPA* anti-citrullinated protein antibodies, *BMI* body mass index, *CRP* c-reactive protein, *DAS* disease activity score, *TJC* tender joint count, *SJC* swollen joint count, *HAQ* health assessment questionnaire, *VAS* visual analogue scale, *SHS* Sharp/van der Heijde score, *JSN* joint space narrowing

None of the predictors showed a statistically significant interaction with the allocated treatment group; therefore, all patients were analyzed together (data not shown). Presence of ≥1 ft erosion (OR 2.32, 95 % CI 1.13; 4.78) was a potential predictor in the univariable model (Table 3). In the multivariable model, presence of ≥1 ft erosion, the combined ACPA–RF variable and DAS appeared statistically significant predictors of OS use. In the final model (Table 3, model 2), the combined ACPA–RF variable was the strongest predictor, with the highest OR for being ACPA+ but RF− (OR 6.14, 95 % CI 1.68; 22.40) and a lower OR for being ACPA+ and RF+ (OR 4.90, 95 % CI 1.85; 12.99), followed by ≥1 ft erosion (OR 2.33, 95 % CI 1.08; 5.03) and baseline DAS (OR 1.77, 95 % CI 1.21; 2.60).

**Table 3:** Logistic regression models to predict orthopaedic shoe use within 10 years of treatment start (*n* = 285)

	Variable	Odds ratio	95 % confidence interval	*p* value	Maximum likelihood *R* ^2^
	Univariate predictors				
	Erosions foot yes/no	2.32	1.13; 4.78	0.022	0.017
	JSN foot yes/no	1.42	0.75; 2.76	0.281	0.004
	Multivariable models				
1	Erosions foot yes/no	2.42	1.10; 5.28	0.027	0.10
	ACPA–RF	Ref.	Ref.	Ref.	
	ACPA− RF+	1.10	0.25; 4.89	0.896	
	ACPA+ RF−	6.43	1.75; 23.55	0.005	
	ACPA+ RF+	4.89	1.82; 13.13	0.002	
	Age	0.98	0.95; 1.01	0.159	
	BMI	1.05	0.96; 1.14	0.296	
	DAS	1.82	1.23; 2.67	0.002	
	Smoking status yes/no	1.29	0.66; 2.51	0.453	
2	Erosions foot yes/no	2.33	1.08; 5.03	0.031	0.090
	ACPA–RF	Ref.	Ref.	Ref.	
	ACPA− RF+	1.12	0.26; 4.93	0.876	
	ACPA+ RF−	6.14	1.68; 22.40	0.006	
	ACPA+ RF+	4.90	1.85; 12.99	0.001	
	DAS	1.77	1.21; 2.60	0.003	

For all binary variables, ‘no’ was used as the reference category. For ACPA–RF, ‘both negative’ was used as the reference category

*JSN* joint space narrowing, *ACPA* anti-citrullinated protein antibodies, *DAS* disease activity score.

## Discussion

This study showed that 57/285 (20 %) recent onset RA patients used OS after 10 years of tightly controlled treatment. This is less than in previous reports on patients with similar disease duration (25 to 30 %) [12, 13], yet higher than expected based on the tightly controlled treatment, with three-monthly joint evaluations including the feet, especially as it may be an underestimation of the actual need for OS [13]. OS in the Netherlands are partially reimbursed for all patients, and OS specialists are easily accessible, which may encourage prescription when medical therapy cannot alleviate symptoms. We investigated whether foot joint damage due to RA can predict OS use, with the underlying idea that with a better understanding of factors that associate with OS use, the need for OS may be prevented in future patients. To the best of our knowledge, this is the first study trying to predict OS use.

We found that OS users had more swollen joints and a trend towards more painful joints of feet and ankles both at baseline and at year 10. Also, joint damage at baseline and joint damage progression from baseline to year 10 was significantly greater in OS users for all damage sub-scores, except for hand erosions. There were no statistically significant differences in DAS (which includes the evaluation of feet and ankles) between the two groups at both time points, yet the DAS was slightly higher in OS users both at baseline and after 10 years. The HAQ of OS users and non-users was comparable at baseline, but after 10 years, a statistically significant and clinically meaningful difference of 0.23 in HAQ score was found between OS users and non-users, with OS users reporting lower functional ability than non-users, despite having similar disease activity. We found that the presence of at least one baseline foot erosion, ACPA positivity with or without RF positivity and a higher DAS at treatment start were independent predictors of OS use after 10 years. RF positivity without ACPA positivity, higher age, BMI, smoking status and foot JSN at baseline were not predictive of future OS use.

This study has some limitations. The date of onset of OS use is unknown, limiting our analysis to potential baseline predictors, in a relatively homogenous patient cohort, due to study inclusion criteria. Nevertheless, it is unlikely that OS were the cause of joint destruction and functional disability [10, 19, 20].

Another limitation of our study is the low response to the 10 years questionnaire. Although most baseline characteristics did not differ between questionnaire responders and non-responders, we do not know what happened with non-responders regarding OS use. Since questionnaire responders were slightly older than non-responders, younger patients appear to be underrepresented, but how this would affect the outcomes is unknown. The low number of OS users (*n* = 57) allowed us to include only a limited number of potential predictors in the logistic regression analysis. Nevertheless, we have found some predictors of OS use that were in line with previously reported data about radiographic damage progression [21].

In conclusion, after 10 years of DAS ≤2.4 targeted treatment, 57/285 (20 %) recent onset RA patients reported to use OS. Presence of foot erosions at treatment start is associated with OS use after 10 years, with ACPA positivity with or without RF positivity and—to a lesser extent—a high baseline DAS contribute additional risk.

## Electronic supplementary material

Below is the link to the electronic supplementary material.

ESM 1 (DOCX 19 kb)
