# Supplementary material for: Erosions in the foot at baseline are predictive of orthopaedic shoe use after 10 years of treat to target therapy in patients with recent onset rheumatoid arthritis
Source: Clin Rheumatol. 2015 Dec 22;35:2101–7. doi: 10.1007/s10067-015-3145-1 (PMC4960271; doi:10.1007/s10067-015-3145-1)
Supplement: Supplementary file 1 — (DOCX 19 kb) [file 10067_2015_3145_MOESM1_ESM.docx]

**Supplementary file**

**Title:** Erosions in the foot at baseline are predictive of orthopaedic shoe use after 10 years of treat to target therapy in patients with recent onset rheumatoid arthritis.

**Journal:** Clinical Rheumatology

**Authors:** Sytske Anne Bergstra MSc^1^, Iris M. Markusse MD^1^, Gülşah Akdemir MD^1^, H. Karel Ronday MD PhD^2^, K. Huub Han MD^3^, Willem F. Lems MD PhD^4,5^, Pit J.S.M. Kerstens MD PhD^5^, Rosaline van den Berg PhD^1^, Robert B.M. Landewé MD PhD^6^, Cornelia F. Allaart MD PhD^1^

**Affiliations**^1^Department of Rheumatology, Leiden University Medical Center, Leiden, The Netherlands
^2^Department of Rheumatology, Haga-Leyenburg Teaching Hospital, The Hague, The Netherlands
^3^Department of Rheumatology, Maasstad Hospital, Rotterdam, The Netherlands
^4^Department of Rheumatology, VU Medical Center, Amsterdam, The Netherlands
^5^Department of Rheumatology, Reade, Amsterdam, The Netherlands
^6^Amsterdam Rheumatology & Immunology Center, Academic Medical Center, Amsterdam, The Netherlands; & Atrium Medical Center Heerlen, The Netherlands

**Corresponding author:** Sytske Anne Bergstra, s.a.bergstra@lumc.nl

| **Supplementary table 1**. Baseline characteristics of patients responding to the question about orthopaedic shoe use (n=285) and patients that did not respond (n=223). | | | | | |
| --- | --- | --- | --- | --- | --- |
|  | **Responders** | | **Non-responders** | | |
|  | **N** |  | **N** |  | **P-value** |
| Age | 285 | 51.3 (12.0) | 223 | 58.4 (14.8) | <0.001 |
| Female (%) | 285 | 67.4 | 223 | 67.7 | 0.934 |
| BMI (kg/m^2^) | 285 | 26.1 (3.7) | 223 | 26.0 (4.7) | 0.779 |
| Smoking (% yes) | 285 | 31.9 | 218 | 39.9 | 0.053 |
| Alcohol drinking (% yes) | 284 | 52.1 | 218 | 42.2 | 0.028 |
| RF positive (%) | 285 | 68.1 | 223 | 60.5 | 0.078 |
| ACPA positive (%) | 277 | 62.1 | 196 | 60.7 | 0.761 |
| CRP [median (range)] | 280 | 21 (1; 238) | 212 | 20 (1; 229) | 0.913 |
| DAS | 285 | 4.4 (0.9) | 223 | 4.4 (0.9) | 0.695 |
| Ritchie articular index | 284 | 14.0 | 223 | 14.2 | 0.789 |
| Total SJC | 284 | 14.6 | 223 | 14.5 | 0.883 |
| TJC foot and ankle joints [median (range)] | 282 | 11 (0; 51) | 222 | 10 (0; 56) | 0.404 |
| SJC foot and ankle joints [median (range)] | 278 | 4 (0; 21) | 221 | 3 (0; 17) | 0.055 |
| ESR [median (range)] | 285 | 36 (2; 143) | 223 | 38 (2; 138) | 0.264 |
| HAQ | 285 | 1.3 (0.6) | 223 | 1.5 (0.7) | 0.020 |
| VAS pain | 284 | 54.0 (21.5) | 222 | 53.3 (22.2) | 0.740 |
| VAS general health | 285 | 51.9 (20.2) | 222 | 53.9 (20.0) | 0.563 |
| VAS disease activity | 284 | 60.0 (22.6) | 222 | 59.9 (22.1) | 0.926 |
| VAS morning stiffness | 284 | 60.2 (24.0) | 222 | 58.8 (23.6) | 0.513 |
| Patients with erosions (%) | 271 | 27.7 | 203 | 35.4 | 0.063 |
| Total SHS [median (range)] | 268 | 2 (0; 35.5) | 201 | 1 (0; 45.5) | 0.285 |
| Bone erosions [median (range)] | 268 | 0 (0; 20.5) | 201 | 0 (0; 9.5) | 0.306 |
| Bone erosions foot [median (range)] | 271 | 0 (0; 20.5) | 203 | 0 (0; 7) | 0.680 |
| Bone erosions hand [median (range)] | 274 | 0 (0; 9.5) | 204 | 0 (0; 6.5) | 0.043 |
| JSN [median (range)] | 269 | 1.5 (0; 21) | 202 | 1 (0; 36) | 0.154 |
| JSN foot [median (range)] | 273 | 0 (0; 15) | 204 | 0 (0; 12) | 0.459 |
| JSN hand [median (range)] | 273 | 1 (0; 34) | 205 | 1 (0; 32) | 0.326 |

Mean (SD) reported if not stated otherwise. RF = rheumatoid factor, ACPA = anti-citrullinated protein antibodies, BMI = body mass index, DAS = disease activity score, TJC = tender joint count, SJC = swollen joint count, ESR = erythrocyte sedimentation rate, HAQ = health assessment questionnaire, VAS = visual analogue scale, CRP = C-reactive protein, SHS = Sharp/van der Heijde score, JSN = joint space narrowing.
